# Immunomodulation by Zearalenone in Carp (*Cyprinus carpio* L.)

**DOI:** 10.1155/2015/420702

**Published:** 2015-09-29

**Authors:** Constanze Pietsch, Ranka Junge, Patricia Burkhardt-Holm

**Affiliations:** ^1^Zurich University of Applied Sciences (ZHAW), Institute of Natural Resource Sciences (IUNR), Gruental, 8820 Waedenswil, Switzerland; ^2^Man-Society-Environment, Department of Environmental Sciences, University Basel, Vesalgasse 1, 4051 Basel, Switzerland; ^3^Department of Biological Sciences, University of Alberta, CW 405 Biological Sciences Building, Edmonton, AB, Canada T6G 2E9

## Abstract

The mycotoxin zearalenone (ZEN) is a frequent contaminant of animal feeds, but its effects on fish have not yet been investigated extensively. In order to fill this gap a feeding trial with juvenile carp was conducted. Three groups of fish were fed feeds contaminated with ZEN at three concentrations (low: 332 *μ*g kg^−1^, medium: 621 *μ*g kg^−1^, and high: 797 *μ*g kg^−1^ feed) for four weeks. Possible reversible effects of ZEN were evaluated by feeding additional groups with the ZEN-contaminated feeds for four weeks, followed by the uncontaminated diet for two weeks. Immune function of isolated leukocytes from head kidney and trunk kidney was assessed using the assessment of NO production, the respiratory burst assay, the chemiluminescence assay, and the measurement of arginase activities. These investigations frequently revealed increased immune responses after exposure of fish to low ZEN concentrations and reduced immune responses after exposure to high mycotoxin concentrations. Moreover, the feeding of the uncontaminated diet for further two weeks did not improve the immune responses in most cases. These results indicate that cellular immune functions in ZEN-contaminated carp are influenced which may be relevant for fish health in aquaculture.

## 1. Introduction

The knowledge on the occurrence of the mycotoxin zearalenone (ZEN), one of the most relevant* Fusarium* toxins, in animal feed including fish feeds is increasing [1, 2], and the evidence on the toxicological effects of ZEN is accumulating. For example, ZEN feeding has already been found to be hepatotoxic and genotoxic in fish [3] and affects haematological parameters and reproduction [3–7]. Most of these effects were attributed to its estrogenic potential [6, 7]; however, also oxidative stress due to ZEN exposure has been identified as a cause of disturbance of fish cell functions [8].

In general, many mycotoxins are reported to be immunotoxic [9]. However, direct effects of ZEN, on the immune system of fish, have not been investigated yet, although a previous publication indicated changes of white blood cell populations in the blood circulation of ZEN-treated carp [3]. Higher vertebrates, domestic as well as laboratory animals, showed impaired immune functions after dietary exposure to ZEN [10–14]. Recently, impairment of both innate and acquired immune responses was shown [15, 16]. Thus, it is reasonable to investigate effects of this mycotoxin in fish.

Impaired immune functions of fish due to dietary mycotoxin exposure may lead to considerable consequences which could contribute to disease problems in aquaculture. The goal of this study is to causally determine the effects of three experimentally ZEN-contaminated diets on innate immune responses of carp (*Cyprinus carpio* L.) since this fish species is important for aquaculture [17].

## 2. Materials and Methods

### 2.1. Chemicals

All chemicals were obtained from Sigma (Buchs, Switzerland) unless indicated otherwise.

### 2.2. Preparation of Feeds

The experimental diets have been prepared without cereal ingredients since natural contamination of grains is known to lead to exposure to more than one mycotoxin. Therefore, only fish meal, blood meal, casein, dextrose, potato starch, vitamins, and minerals were used for feed preparation as described previously [3]. Prior to the preparation of pellets in a pelletizer (L 14-175, Amandus Kahl, Reinbek, Germany) zearalenone (ZEN, dissolved in ethanol; purity > 99%, lot number 041M4054V) was added at three different concentrations (low dose: 332 *μ*g kg^−1^, medium dose: 621 *μ*g kg^−1^, and high dose: 797 *μ*g kg^−1^ final feed, resp., determined by HPLC with fluorescence detection after a cleanup with IAC (immunoaffinity column, ZearalaTest, Vicam, Klaus Ruttmann, Hamburg, Germany) as described elsewhere [18]) to the feed ingredients whereas the control diet was not supplied with ZEN. Consequently, no ZEN was detected in the control diet [3]. The final diets were isonitrogenous and isocaloric [3] and were stored at 4°C until use.

### 2.3. Exposure of Fish

Mirror carp (Aischgründer strain, Bavaria, Germany) of 12–16 cm in length (weighting 27.13 ± 1.13 g, mean ± SEM) were used for the feeding trial. All fish were kept at a 16 h light/8 h dark photoperiod at 24.9 ± 0.4°C (mean ± SD) in a flow-through system providing approximately 6 L of fresh water per h for each tank. Three weeks prior to the start of the experiments, the carp were sorted into the four feeding groups and received the uncontaminated experimental diet. Each feeding group included four tanks of 54 L containing 6 fish each. Water parameters (dissolved oxygen, pH, temperature, and conductivity) were monitored for each tank at least three times a week and were within optimal range as published previously [3]. During the experiments fish were fed the experimentally ZEN-contaminated diets for four weeks while the control group received the uncontaminated feed at a feeding intensity of 3% of body weight per day. During the experimental phase, uptake of the experimental diets was observed in all groups within less than 30 min after offering the feed. After four weeks of feeding, two tanks per feeding group were sampled. In addition, two additional tanks per feeding group were fed the uncontaminated diet for two more weeks before the final sampling in order to evaluate possible recovery from ZEN feeding. After the indicated time of exposure fish were killed by a blow on the head, and the immune organs were surgically removed. The experimental procedures have been approved by the Cantonal veterinarian authorities of Basel-Stadt (Switzerland) under the permission number 2410.

### 2.4. Culture of Immune Cells and Exposure to Stimulants

Innate immune responses were determined in primary cell cultures from carp head and trunk kidneys prepared as described by Pietsch et al. [19]. Stimulation of nitric oxygen (NO) production was achieved by addition of 30 *μ*g mL^−1^ bacterial lipopolysaccharide (LPS from* E. coli*, serotype O111:B4) to wells followed by incubation of cells at 25°C and 5% CO_2_ for 96 h. Subsequently, NO production was analyzed using the Griess reagent as described by Pietsch et al. [20]. Arginase activity was measured after 24 h with and without addition of forskolin as described previously [19]. All experiments were run in 3 independent replicates.

### 2.5. Measurement of Cell Viability and Respiratory Burst Activity

In parallel to incubations for assessing NO production, cell viability after exposure to ZEN was measured using neutral red (3-amino-7-dimethylamino-2-methylphenazine hydrochloride, NR) uptake based on the method described by Borenfreund and Puerner [21] in order to evaluate membrane integrity and lysosomal function. Therefore, a working solution containing 0.0025% NR was prepared in Earle's medium and added to cells for 3 h. Thereafter, cells were washed twice with sterile Earle's medium, lysed in 50 *μ*L ethanol containing 2% acetic acid, and optical densities were analyzed at a wavelength of 540 nm (Infinite M200, Tecan Group Ltd., Männedorf, Switzerland).

Respiratory burst activity was analyzed with the nitroblue tetrazolium (NBT) assay as has been described by Chung and Secombes [22] with some modifications. Accordingly, leukocytes were cultured at 25°C and 5% CO_2_ for 24 and 96 h and incubated for 1 h with 1 mg NBT mL^−1^ culture medium (RPMI medium without phenol red, containing bicarbonate and 4-(2-hydroxyethyl)piperazine-1-ethanesulfonic acid (HEPES) supplemented with 10% sterile distilled water, penicillin (100 U mL^−1^), streptomycin (100 *μ*g mL^−1^), and 2 mM L-glutamine) with and without stimulation by addition of 0.24 *μ*g mL^−1^ phorbol myristate acetate (PMA). After discarding the supernatant, cells were fixed using 70% methanol and the air-dried plates were incubated with 50 *μ*L dimethyl sulfoxide (DMSO) and 50 *μ*L potassium hydroxide (KOH) to solubilize the formazan. Absorbance at 620 nm was measured spectrophotometrically in duplicate with a microplate reader (Infinite M200, Tecan Group Ltd., Männedorf, Switzerland) using DMSO/KOH alone as blank.

Chemiluminescence was measured according to Verlhac et al. [23] and Lundén et al. [24] in leukocyte cultures after 24 h* ex vivo *using the luminescence mode of the Infinite M200 (Tecan Group Ltd., Männedorf, Switzerland). Therefore, the cell culture medium was replaced by 50 *μ*L Earle's medium per well, followed by addition of 50 *μ*L luminol (5-amino-2,3-dihydro-1,4-phthalazinedione) solution or 50 *μ*L lucigenin (bis-N-methylacridinium nitrate) solution at final concentrations of 0.05 and 0.1 *μ*mol per well in Earle's medium, respectively. Luminol allows estimation of myeloperoxidase-dependent activities of activated phagocytes, which reflects a common host defense mechanism [25], but this dye also detects reactive oxygen species, such as hydrogen peroxide, hydroxyl radicals, and singlet oxygen [26]. Lucigenin can be used for detection of extracellular superoxide anions [26]. Initially, background luminescence of leukocytes was read. Afterwards, cells were stimulated by addition of 25 *μ*L Earle's medium containing opsonized zymosan at a final concentration of 0.026 mg per well. The latter had been prepared by mixing 100 mg zymosan with 0.85% sodium chloride solution followed by addition of 4.5 mL freshly prepared carp serum (prepared from blood that was drawn from the caudal vein and centrifuged for 5 min at 3,000 g (Centrifuge 5415R, Eppendorf, Basel, Switzerland)). This mixture was then incubated for 20 min at 37°C. Afterwards, the zymosan had been washed twice with the sodium chloride solution and aliquots had been stored at −80°C until use. Luminescence by stimulated leukocytes was recorded for 70 min at 22°C and calculated as relative luminescence units (RLU) per mg protein. Therefore, protein contents in all wells were assessed by the BCA Protein kit (Sigma) according to the manufacturer's protocol after lysis of cell using Triton X-100 at a final concentration of 0.1%.

### 2.6. Statistics

Effects of treatments were determined by comparison of treatment groups to controls using the Mann-Whitney *U* test. Successful stimulation of immune cells within a treatment group was analyzed using Friedman test followed by Wilcoxon test (SPSS 9.0 for Windows). Differences between treatment groups were considered statistically significant when *P* < 0.05.

## 3. Results

**Figure 1:**
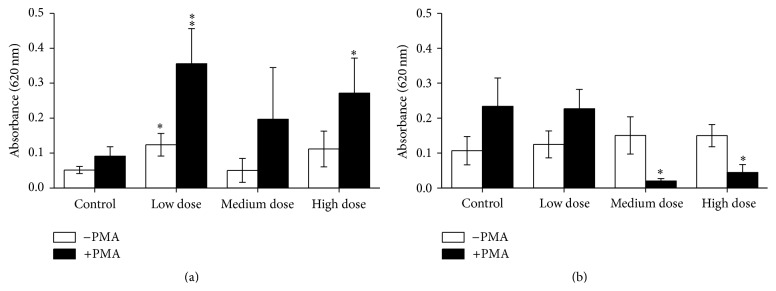
NBT test after 24 h* in vitro* culture in head kidney cells. Respiratory burst after 24 h of* in vitro* culture of head kidney cells of fish after 4 weeks of feeding ZEN (a) and with additional 2 weeks of recovery (b) assayed by means of conversion of NBT with and without additional stimulation by PMA for 90 min measured by absorbance at 620 nm, *n* = 6; mean ± SEM; *∗* = difference to controls at *P* < 0.05; *∗∗* = difference to controls at *P* < 0.01.

**Figure 2:**
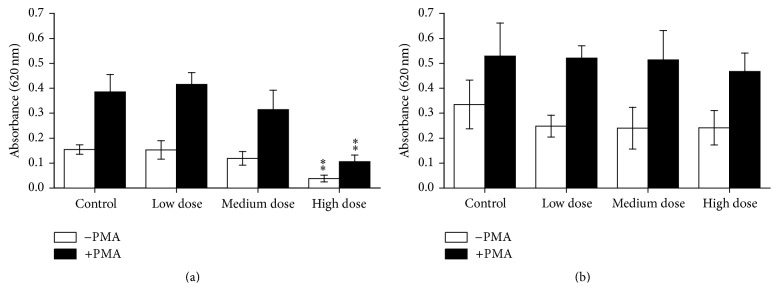
NBT test after 24 h* in vitro* culture in trunk kidney cells. Respiratory burst after 24 h of* in vitro* culture of trunk kidney cells of fish after 4 weeks of feeding ZEN (a) and with additional 2 weeks of recovery (b) assayed by means of conversion of NBT with and without stimulation by PMA for 90 min measured as an absorbance at 620 nm, *n* = 6; mean ± SEM; *∗∗* = difference to controls at *P* < 0.01.

**Figure 3:**
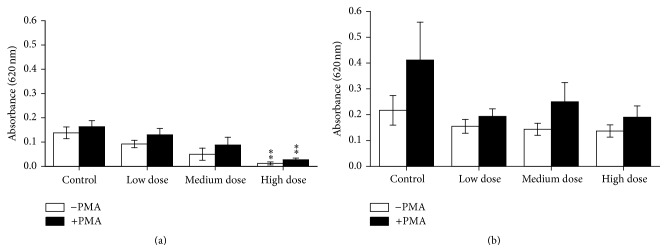
NBT after 96 h* in vitro* culture in trunk kidney cells. Respiratory burst after 96 h of* in vitro* culture of trunk kidney cells of fish after 4 weeks of feeding ZEN (a) and with additional 2 weeks of recovery (b) assayed by means of conversion of NBT with and without stimulation by PMA for 90 min measured as an absorbance at 620 nm, *n* = 6; mean ± SEM; *∗∗* = difference to controls at *P* < 0.01.

**Figure 4:**
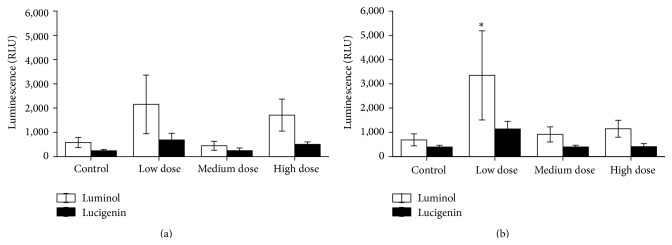
Chemiluminescence after 24 h* in vitro* culture in head kidney cells. Chemiluminescence (calculated per mg cell protein) of head kidney cells derived from fish after 4 weeks of feeding ZEN (a) and with additional 2 weeks of recovery (b) after 24 h of* in vitro* culture of leukocytes followed by stimulation with opsonized zymosan (for 70 min) measured with the substrates luminol and lucigenin, *n* = 6; mean ± SEM; *∗* = difference to controls at *P* < 0.05.

**Figure 5:**
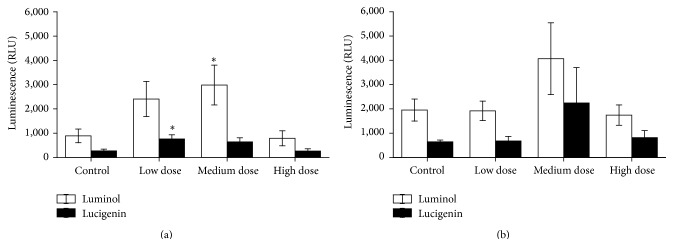
Chemiluminescence after 24 h* in vitro* culture in trunk kidney cells. Chemiluminescence (calculated per mg cell protein) of trunk kidney cells derived from fish after 4 weeks of feeding ZEN (a) and with additional 2 weeks of recovery (b) after 24 h of* in vitro* culture of leukocytes followed by stimulation with opsonized zymosan (for 70 min) measured with the substrates luminol and lucigenin, *n* = 6; mean ± SEM; *∗* = difference to controls at *P* < 0.05.

Innate immune responses of the experimental fish were influenced by ZEN feeding. This included NBT conversion after 24 h of* in vitro* culture that was increased in PMA-stimulated and unstimulated head kidney leukocytes from the low dose ZEN group which was also observed in PMA-stimulated leukocytes from fish fed the high dose ZEN diet (Figure 1(a)). Two weeks of recovery led to reduced respiratory bursts in leukocytes from head kidneys of fish previously fed the feeds contaminated with the medium and high ZEN dose compared with control fish whereas the fish from the low dose ZEN group no longer showed an influence of ZEN feeding (Figure 1(b)). In trunk kidney leukocytes reduced NBT conversion in stimulated and unstimulated cells was only observed in fish from the high dose ZEN group which was no longer detectable after two weeks of recovery from ZEN feeding (Figure 2). Since cell isolation from trunk kidneys yielded more cells further exposures and NBT assays after 96 h were possible. Similarly to the previous results,* in vitro* culture of trunk kidney-derived leukocytes for 96 h showed decreased respiratory bursts with and without PMA stimulation in fish of the high dose group which was no longer observable after the recovery phase (Figure 3). In accordance to the measurements of NBT conversion the assessment of chemiluminescence by luminol showed increases in head kidney-derived leukocytes of fish fed the low ZEN dose although a significant difference to control fish was only observed after the recovery phase (Figure 4). In trunk kidney leukocytes increased chemiluminescence by luminol was observed in the fish fed the medium dose ZEN diet and increased chemiluminescence by lucigenin in the low dose ZEN-fed fish (Figure 5). Due to high variability in the data these differences were no longer identified in trunk kidney leukocytes from fish after the recovery phase. Moreover, measurements of chemiluminescence after 96 h of* in vitro* culture of leukocytes showed values close to background signals and differences between fish could no longer be detected (data not shown).

**Figure 6:**
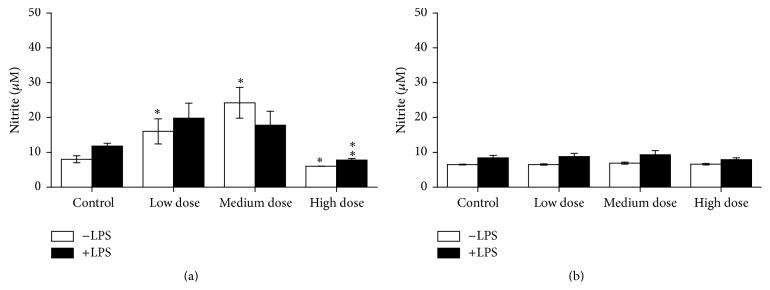
NO test in head kidney cells. NO production by isolated leukocytes from head kidney of experimental fish after 4 weeks of feeding (a) and with additional 2 weeks of recovery (b), *n* = 6; after incubation with and without LPS for 96 h, mean ± SEM; *∗* = difference to controls at *P* < 0.05; *∗∗* = difference to controls at *P* < 0.01.

**Figure 7:**
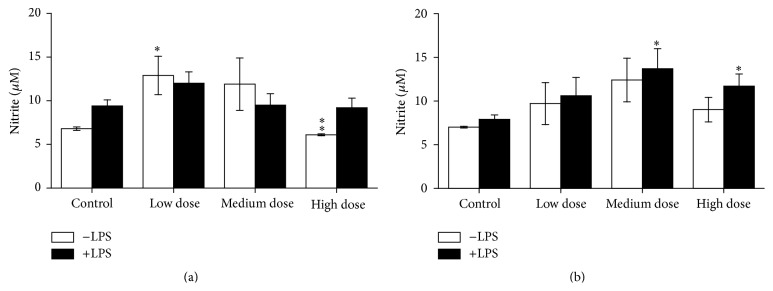
NO test in trunk kidney cells. NO production in trunk kidney cells after incubation with and without LPS for 96 h from experimental fish after 4 weeks of feeding (a) and with additional 2 weeks of recovery (b), *n* = 6; mean ± SEM, difference to controls at *P* < 0.05; *∗∗* = difference to controls at *P* < 0.01.

Compared to control fish, NO production of unstimulated head kidney leukocytes was increased in fish fed the low and the medium ZEN dose feeds but reduced in fish from the high dose ZEN group (Figure 6(a)). The latter was also observed in LPS-stimulated leukocytes. NO production showed only low values after the two-week recovery phase (Figure 6(b)). In unstimulated trunk kidney leukocytes increased NO production in fish of the low dose ZEN group and reduced values in fish fed the highly ZEN-contaminated diet have also been observed (Figure 7(a)). However, after the recovery phase fish, previously fed the medium and high ZEN doses, showed increased NO production of LPS-stimulated leukocytes compared with the control fish (Figure 7(b)).

**Figure 8:**
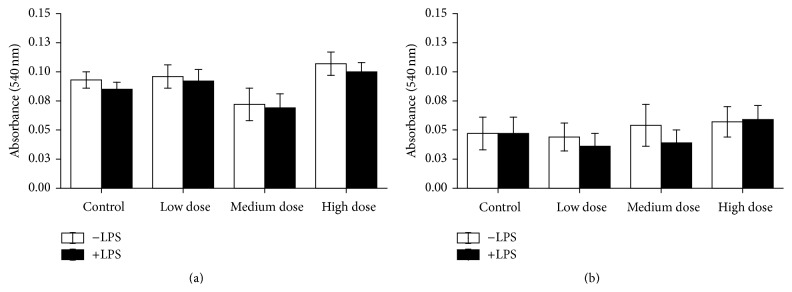
NR test after the NO test in head kidney cells. Cell vitality after 96 h of incubation with and without LPS in* in vitro* cultures of head kidney cells of fish after 4 weeks of ZEN feeding (a) and additional 2 weeks of recovery (b), *n* = 6, mean ± SEM; difference to controls at *P* < 0.05.

**Figure 9:**
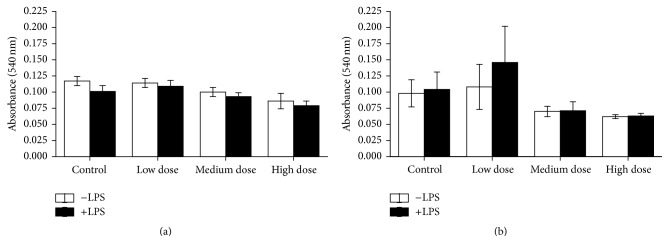
NR test after the NO test in trunk kidney cells. Cell vitality after 96 h of* in vitro* culture of trunk kidney cells of fish after 4 weeks of feeding ZEN (a) and with additional 2 weeks of recovery (b) assayed by means of uptake of neutral red measured by absorption at 540 nm, *n* = 6; mean ± SEM; difference to controls at *P* < 0.05.

Cell viability showed no significant differences due to ZEN feeding neither in head kidney-derived cells nor in trunk kidney leukocytes (Figures 8 and 9).

**Figure 10:**
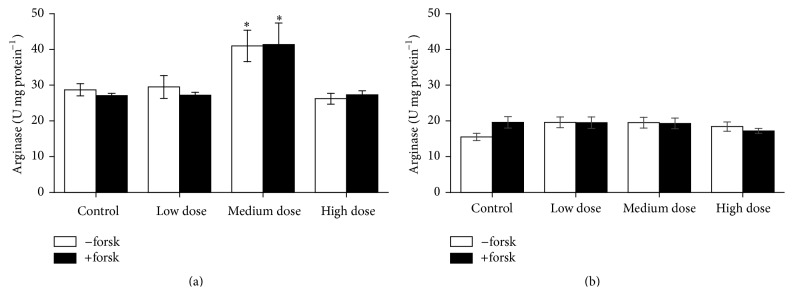
Arginase activity in head kidney cells. Arginase activity in leukocytes derived from head kidney after 4 weeks of feeding ZEN at different concentration levels for 4 weeks (a) and with additional 2 weeks of recovery (b), stimulated for 24 h with 1 *μ*M forskolin compared to unstimulated cells, mean ± SEM; *∗* = difference to controls at *P* < 0.05.

**Figure 11:**
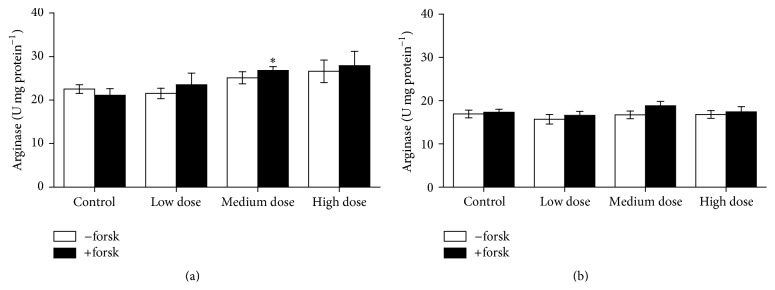
Arginase activity in trunk kidney cells. Arginase activity in leukocytes derived from trunk kidney after 4 weeks of feeding ZEN at different concentration levels for 4 weeks (a) and with additional 2 weeks of recovery (b), stimulated for 24 h with 1 *μ*M forskolin compared to unstimulated cells, mean ± SEM; *∗* = difference to controls at *P* < 0.05.

Arginase activity in head kidney and trunk kidney leukocytes was found to be increased in fish fed the medium dose ZEN feed, but enzyme activities after the recovery phase returned to control levels (Figures 10 and 11). However, arginase stimulation with forskolin did not significantly increase enzyme activity as it commonly does [19, 20].

## 4. Discussion

As published recently contamination of experimental diets containing the low mycotoxin dose [3] was comparable to ZEN values that can be found in commercially available feeding stuffs [2]. The two higher concentrations exceed the values that have been found in commercial fish feed up to now but still showed lower concentrations than the current guidance value by the European Commission of 2 mg kg^−1^ feed [27].

Toxicity of ZEN has mostly been investigated with respect to its possible endocrine and developmental impairment since it is a potent natural estrogen [6, 7]. However many more toxic actions of ZEN have been identified in vertebrates including fish. These actions include hepatotoxic and genotoxic effects and changes of blood parameters in fish [3, 6, 27]. However, immunotoxicity of ZEN has not yet been investigated in fish.

Similar to higher vertebrates the effects of ZEN on fish cells have been related to oxidative stress [8]; thus, it was not surprising that the respiratory burst assay showed differences between ZEN-treated fish and control group. ZEN increased the respiratory burst by leukocytes at low ZEN concentration in the diet and showed immunosuppressive effects at high ZEN concentrations. The effects on arginase activities on head and trunk kidney cells and effects on the respiratory burst and chemiluminescence in trunk kidney cells were reversible by feeding the ZEN-exposed fish for further two weeks with the uncontaminated control feed. However, effects of ZEN on immune functions in trunk kidney cells and on NO production in head and trunk kidney leukocytes have also been observed after recovery for two weeks. Thus, it remains to be evaluated whether a prolonged recovery phase would have reversed the remaining effects of ZEN feeding. The observed effects on cellular immune responses were obviously not related to a reduced viability during the* in vitro* culture of cells which was assessed by NR uptake after the NO assay.

Differences in white blood cell counts, and increased granulocytes coupled with reduced monocyte numbers in blood circulation have been observed in ZEN-treated carp after four weeks of ZEN feeding [3]. These differential effects on leukocyte populations lead to effects on cellular immune responses that have already been suggested for ZEN-fed sheep [28]. Since the two dyes that have been used for assessment of chemiluminescence by leukocytes measure slightly different endpoints, they also may indicate the involvement of different leukocyte populations. Macrophages of rainbow trout are considered to be myeloperoxidase-negative or only weakly positive [29, 30]. If the same is true for macrophages of carp the increased luminol-dependent chemiluminescence in ZEN-treated fish may be due to the response of granulocytes which have been found to be increased in blood circulation in carp fed the medium and the high ZEN diet while monocyte numbers have been reported to be decreased by these treatments [3]. The extracellular production of superoxide anions as measured by lucigenin luminescence was only increased in trunk kidney cells of fish treated with the low dose ZEN diet which suggests that this pathway for production of reactive oxygen species is less sensitive to ZEN treatment.

In general, mycotoxins such as ZEN exhibit their cytotoxic potential primarily on liver tissue of mammals [31, 32]. However, also in the intestine, in thymocytes, and in splenocytes of rodents oxidative stress and immunological changes have been observed due to ZEN application [12, 14]. In swine, ZEN led to altered immune responses* in vitro* including cytotoxicity and changes of cytokine expression [10, 11]. Similarly, splenic lymphocytes from chicken showed altered cytokine expression due to ZEN treatment [13]. This was also confirmed by studies using swine and rats [15, 33]. Also for the gut-associated immune system, differential effects of ZEN on proinflammatory and anti-inflammatory cytokines in swine have been described [34]. From these studies it was concluded that ZEN-treated animals are incapable of inducing adequate immune responses which is highly relevant for disease problems in animal husbandry. This is probably also true for husbandry of fish in aquaculture. However, the exact cellular mechanism of action of ZEN on fish immune cells still remains to be investigated and further research is needed to evaluate possible effects of this mycotoxin on cytokine expression patterns in fish.

## 5. Conclusions

While low dietary ZEN concentrations activate certain cellular immune functions, the exposure to higher ZEN doses impaired the innate immune responses of carp although the ZEN concentrations in the experimental feeds still remained far below the current maximum allowable levels recommended by the European Commission (EC, 2006). This stays in contrast to the results of several studies on higher vertebrates which showed effects on immune parameters, antioxidative enzymes, and growth parameters due to ZEN feeding at dietary concentrations ranging from 2 mg per kg to up to 800 mg per kg [12–15, 35, 36]. Therefore, it can be concluded from the present study that fish are very sensitive to dietary ZEN concentrations and that ZEN contamination in fish feeds probably poses even higher threats for fish health in aquaculture than hitherto expected.

## References

[1] Santos G. A., Rodrigues I., Naehrer K., Encarnacao P. (2010). Mycotoxins in aquaculture: occurrence in feed components and impact on animal performance. *Aquaculture Europe*.

[2] Pietsch C., Kersten S., Burkhardt-Holm P., Valenta H., Dänicke S. (2013). Occurrence of deoxynivalenol and zearalenone in commercial fish feed. An initial study. *Toxins*.

[3] Pietsch C., Kersten S., Valenta H. (2015). Effects of dietary exposure to zearalenone (ZEN) on carp (*Cyprinus carpio* L.). *Toxins*.

[4] Johns S. M., Denslow N. D., Kane M. D., Watanabe K. H., Orlando E. F., Sepúlveda M. S. (2011). Effects of estrogens and antiestrogens on gene expression of fathead minnow (*Pimephales promelas*) early life stages. *Environmental Toxicology*.

[5] Schwartz P., Thorpe K. L., Bucheli T. D., Wettstein F. E., Burkhardt-Holm P. (2010). Short-term exposure to the environmentally relevant estrogenic mycotoxin zearalenone impairs reproduction in fish. *Science of the Total Environment*.

[6] Woźny M., Brzuzan P., Gusiatin M., Jakimiuk E., Dobosz S., Kuźmiński H. (2012). Influence of zearalenone on selected biochemical parameters in juvenile rainbow trout (*Oncorhynchus mykiss*). *Polish Journal of Veterinary Sciences*.

[7] Bakos K., Kovács R., Staszny Á. (2013). Developmental toxicity and estrogenic potency of zearalenone in zebrafish (*Danio rerio*). *Aquatic Toxicology*.

[8] Pietsch C., Noser J., Wettstein F. E., Burkhardt-Holm P. (2014). Unraveling the mechanisms involved in zearalenone-mediated toxicity in permanent fish cell cultures. *Toxicon*.

[9] Bondy G. S., Pestka J. J. (2000). Immunomodulation by fungal toxins. *Journal of Toxicology and Environmental Health Part B: Critical Reviews*.

[10] Marin D. E., Taranu I., Burlacu R., Tudor D. S. (2010). Effects of zearalenone and its derivatives on the innate immune response of swine. *Toxicon*.

[11] Marin D. E., Taranu I., Burlacu R. (2011). Effects of zearalenone and its derivatives on porcine immune response. *Toxicology in Vitro*.

[12] Abbès S., Salah-Abbès J. B., Sharafi H., Noghabi K. A., Oueslati R. (2012). Interaction of *Lactobacillus plantarum* MON03 with Tunisian Montmorillonite clay and ability of the composite to immobilize Zearalenone *in vitro* and counteract immunotoxicity *in vivo*. *Immunopharmacology and Immunotoxicology*.

[13] Wang Y. C., Deng J. L., Xu S. W. (2012). Effects of zearalenone on IL-2, IL-6, and IFN-gamma mRNA levels in the splenic lymphocytes of chickens. *The Scientific World Journal*.

[14] Liu M., Gao R., Meng Q. (2014). Toxic effects of maternal zearalenone exposure on intestinal oxidative stress, barrier function, immunological and morphological changes in rats. *PLoS ONE*.

[15] Choi B.-K., Cho J.-H., Jeong S.-H. (2012). Zearalenone affects immune-related parameters in lymphoid organs and serum of rats vaccinated with porcine parvovirus vaccine. *Toxicological Research*.

[16] Hueza I. M., Raspantini P. C. F., Raspantini L. E. R., Latorre A. O., Górniak S. L. (2014). Zearalenone, an estrogenic mycotoxin, is an immunotoxic compound. *Toxins*.

[17] FAO (2012). *The State of World Fisheries and Aquaculture*.

[18] Brezina U., Valenta H., Rempe I., Kersten S., Humpf H.-U., Dänicke S. (2014). Development of a liquid chromatography tandem mass spectrometry method for the simultaneous determination of zearalenone, deoxynivalenol and their metabolites in pig serum. *Mycotoxin Research*.

[19] Pietsch C., Neumann N., Preuer T., Kloas W. (2011). *In vivo* treatment with progestogens causes immunosuppression of carp *Cyprinus carpio* leucocytes by affecting nitric oxide production and arginase activity. *Journal of Fish Biology*.

[20] Pietsch C., Vogt R., Neumann N., Kloas W. (2008). Production of nitric oxide by carp (*Cyprinus carpio* L.) kidney leukocytes is regulated by cyclic 3′,5′-adenosine monophosphate. *Comparative Biochemistry and Physiology—A Molecular and Integrative Physiology*.

[21] Borenfreund E., Puerner J. A. (1985). Toxicity determined *in vitro* by morphological alterations and neutral red absorption. *Toxicology Letters*.

[22] Chung S., Secombes C. J. (1988). Analysis of events occurring within teleost macrophages during the respiratory burst. *Comparative Biochemistry and Physiology Part B: Comparative Biochemistry*.

[23] Verlhac V., Obach A., Gabaudan J., Schüep W., Hole R. (1998). Immunomodulation by dietary vitamin C and glucan in rainbow trout (*Oncorhynchus mykiss*). *Fish & Shellfish Immunology*.

[24] Lundén T., Lilius E.-M., Bylund G. (2002). Respiratory burst activity of rainbow trout (*Oncorhynchus mykiss*) phagocytes is modulated by antimicrobial drugs. *Aquaculture*.

[25] Klebanoff S. J. (2005). Myeloperoxidase: friend and foe. *Journal of Leukocyte Biology*.

[26] Caldefie-Chézet F., Walrand S., Moinard C., Tridon A., Chassagne J., Vasson M.-P. (2002). Is the neutrophil reactive oxygen species production measured by luminol and lucigenin chemiluminescence intra or extracellular? Comparison with DCFH-DA flow cytometry and cytochrome c reduction. *Clinica Chimica Acta*.

[27] European Commission (2006). Commission Recommendation (2006/576/EC) of 17 August 2006 on the presence of deoxynivalenol, zearalenone, ochratoxin A, T-2 and HT-2 and fumonisins in products intended for animal feeding. *Official Journal of the European Union*.

[28] Kostro K., Gajecka M., Lisiecka U. (2011). Subpopulation of lymphocytes CD4+ and CD8+ in peripheral blood of sheep with zearalenone mycotoxicosis. *Bulletin of the Veterinary Institute in Pulawy*.

[29] Płytycz B., Flory C. M., Galvan I., Bayne C. J. (1989). Leukocytes of rainbow trout (*Oncorhynchus mykiss*) phonephros: cell types producing superoxide anion. *Developmental and Comparative Immunology*.

[30] Secombes C. J., Iwama G., Nakanishi T. (1996). The non-specific immune system: cellular defenses. *The Fish Immune System: Organism, Pathogen, Environment*.

[31] Ding X., Lichti K., Staudinger J. L. (2006). The mycoestrogen zearalenone induces CYP3A through activation of the pregnane X receptor. *Toxicological Sciences*.

[32] Zhou C., Zhang Y., Yin S., Jia Z., Shan A. (2015). Biochemical changes and oxidative stress induced by zearalenone in the liver of pregnant rats. *Human and Experimental Toxicology*.

[33] Pistol G. C., Braicu C., Motiu M. (2015). Zearalenone mycotoxin affects immune mediators, MAPK signalling molecules, nuclear receptors and genome-wide gene expression in pig spleen. *PLoS ONE*.

[34] Obremski K. (2014). Changes in Th1 and Th2 cytokine concentrations in ileal Peyer's patches in gilts exposed to zearalenone. *Polish Journal of Veterinary Sciences*.

[35] Kiessling K. H. (1982). The effect of zearalenone on growth rate, organ weight and muscle fibre composition in growing rats. *Acta Pharmacologica et Toxicologica*.

[36] Allen N. K., Mirocha C. J., Weaver G., Aakhus-Allen S., Bates F. (1981). Effects of dietary zearalenone on finishing broiler chickens and young turkey poults. *Poultry Science*.

